# The Host Peritoneal Cavity Harbors Prominent Memory Th2 and Early Recall Responses to an Intestinal Nematode

**DOI:** 10.3389/fimmu.2022.842870

**Published:** 2022-03-28

**Authors:** Ivet A. Yordanova, Karsten Jürchott, Svenja Steinfelder, Katrin Vogt, Ulrike Krüger, Anja A. Kühl, Birgit Sawitzki, Susanne Hartmann

**Affiliations:** ^1^ Institute of Immunology, Center for Infection Medicine, Freie Universität Berlin, Berlin, Germany; ^2^ Berlin Institute of Health Center for Regenerative Therapies (BCRT), Charité Universitätsmedizin Berlin, Berlin, Germany; ^3^ Max-Delbrück Center for Molecular Medicine, Berlin, Germany; ^4^ Institute of Medical Immunology, Charité Universitätsmedizin Berlin, Berlin, Germany; ^5^ Core Unite Genomics, Berlin Institute of Health (BIH), Berlin, Germany; ^6^ Charité Universitätsmedizin Berlin, Corporate Member of Freie Universität Berlin und Humboldt-Universität zu Berlin, iPATH.Berlin, Core Unit for Immunopathology for Experimental Models, Berlin, Germany

**Keywords:** memory Th2 cell, intestinal nematode, peritoneal cavity, dendritic cells, eosinophils, Ox40, recall response

## Abstract

Intestinal parasitic nematodes affect a quarter of the world’s population, typically eliciting prominent effector Th2-driven host immune responses. As not all infected hosts develop protection against reinfection, our current understanding of nematode-induced memory Th2 responses remains limited. Here, we investigated the activation of memory Th2 cells and the mechanisms driving early recall responses to the enteric nematode *Heligmosomoides polygyrus* in mice. We show that nematode-cured mice harbor memory Th2 cells in lymphoid and non-lymphoid organs with distinct transcriptional profiles, expressing recirculation markers like CCR7 and CD62-L in the mesenteric lymph nodes (mLN), and costimulatory markers like Ox40, as well as tissue homing and activation markers like CCR2, CD69 and CD40L in the gut and peritoneal cavity (PEC). While memory Th2 cells persist systemically in both lymphoid and non-lymphoid tissues following cure of infection, peritoneal memory Th2 cells in particular displayed an initial prominent expansion and strong parasite-specific Th2 responses during early recall responses to a challenge nematode infection. This effect was paralleled by a significant influx of dendritic cells (DC) and eosinophils, both also appearing exclusively in the peritoneal cavity of reinfected mice. In addition, we show that within the peritoneal membrane lined by peritoneal mesothelial cells (PeM), the gene expression levels of cell adhesion markers VCAM-1 and ICAM-1 decrease significantly in response to a secondary infection. Overall, our findings indicate that the host peritoneal cavity in particular harbors prominent memory Th2 cells and appears to respond directly to *H. polygyrus* by an early recall response *via* differential regulation of cell adhesion markers, marking the peritoneal cavity an important site for host immune responses to an enteric pathogen.

**Graphical Abstract:**
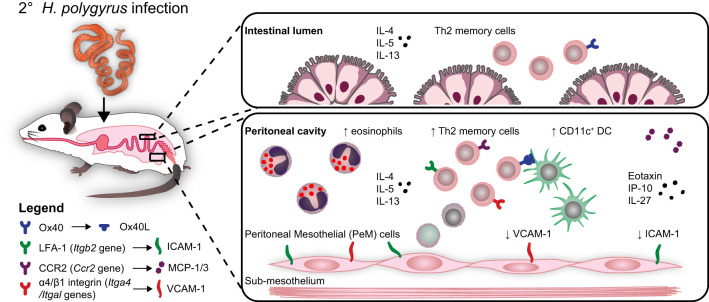
Freie Universität Berlin owns the copyright to the graphical abstract.

## Introduction

Following antigen exposure during a primary infection, the host immune system typically initiates molecular and cellular processes of immunological memory, reliant on functional long-lived CD4^+^ or CD8^+^ memory T cells (1, 2). These memory T cells normally seed lymphoid and non-lymphoid tissues as either tissue-resident memory (T_RM_), recirculating effector memory (T_EM_) or central memory T cells (T_CM_). While T_CM_ cells with a typical CD4^+^CD44^+^CD62-L^+^ phenotype primarily recirculate between blood and secondary lymphoid organs, CD4^+^CD44^+^CD62-L^-^ T_EM_ migrate between blood and non-lymphoid tissues. In contrast, CD4^+^CD44^+^CD62-L^-^ T_RM_ express various tissue homing and retention markers like CD49d, CD69 or CD103 and take up residence in mucosal barrier tissues (3, 4). Upon pathogen re-exposure, T_RM_ cells are thus able to coordinate a faster, localized recall immune response to re-infection.

Humans typically develop only limited protective immunity against re-infection with parasitic nematodes, evidenced by commonly occurring re-infections in endemic areas (5, 6). Nevertheless, some evidence exists of the development of effective, albeit partial protective immunity against nematodes with age (7, 8). In both humans and animals, the signature effector responses to a primary nematode infection rely on Gata3^+^ effector Th2 cells, IgE and IgG antibody production, alternatively-activated macrophages (AAM) and suppressive Foxp3^+^ regulatory T cells (Treg) (9–12). We have shown that mice infected with the strictly intestinal nematode *Heligmosomoides polygyrus* harbor prominent tissue-resident memory Th2 cells in various locations, including the small intestinal lamina propria (siLP) and the peritoneal cavity (PEC) 8 weeks post-cure of infection (4). These cells secrete high levels of IL-4, IL-5 and IL-13 cytokines upon *in vitro* restimulation, while upon adoptive transfer they reduce female worm fecundity, indicative of a protective role (4). Functional memory Th2 cells also accumulate in the lung following infection with the tissue-migratory nematode *Nippostrongylus brasiliensis* in mice, conferring protection to re-infection in an IL-4- and eosinophil-dependent manner (13, 14). *H. polygyrus*-induced activated T cells in the lung are also associated with cross-protection to a *N. brasiliensis* challenge infection (15), highlighting the systemic distribution and cross-protective potential of memory T cells against unrelated nematodes (16).

In light of our previous findings on the presence of highly functional peritoneal memory Th2 cells in *H. polygyrus*-cured mice, the local mechanisms driving immune cell recruitment and activation in the peritoneal cavity during an otherwise strictly intestinal infection remain unclear. Thus, here we focused on characterizing the transcriptional profiles of nematode-induced peritoneal memory Th2 cells, their activation during early recall responses to *H. polygyrus* and local mechanisms in the peritoneal compartment potentially influencing immune cell recruitment and activation following cure and reinfection.

## Results

### Nematode-Cured Mice Harbor Long-Lived, Phenotypically Distinct CD4^+^ T Cells in Lymphoid and Non-Lymphoid Organs

**Figure 1:**
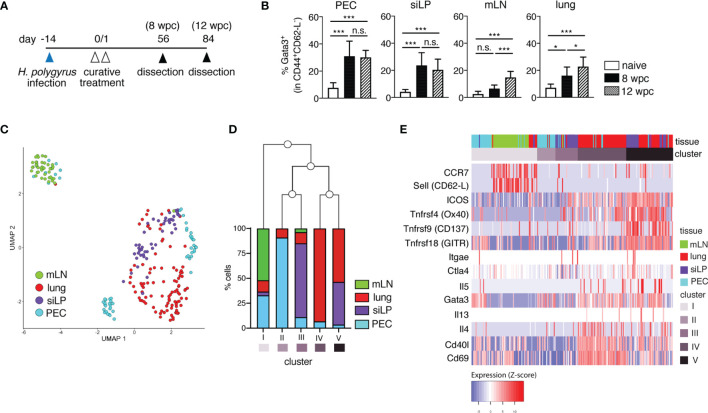
Memory T cells persist long-term and display distinct transcriptional profiles in lymphoid versus non-lymphoid tissues following primary nematode infection. **(A)** Experimental set-up. Mice were infected *via* oral inoculation with 225 infective L3 *H. polygyrus* larvae and were cured of infection 14 days later *via* oral administration of 2 mg/mouse pyrantel pamoate anthelminthic on two consecutive days. The mice were then allowed to rest and were sacrificed 8- and 12-weeks post-cure. **(B)** Frequencies of memory Th2 cells in peritoneal cavity (PEC), small intestinal lamina propria (siLP), mesenteric lymph nodes (mLN) and lung of naïve and cured mice. **(C)** UMAP of single-cell RNA-seq data of sorted CD4^+^ T cells from PEC, siLP, mLN and lungs of mice sacrificed at 8 weeks post-cure. **(D)** Bar graph illustrating the five different clusters defined by hierarchical clustering of hyper-variable expressed genes in the single cell transcriptomes and the frequencies of cells from each tissue contributing to each cluster. **(E)** Heatmap representation of selected marker genes. The order of the columns derived from the hierarchical clustering of the hyper-variable expressed genes and the order of the rows was predefined by the authors. Different colours correspond to the scaled expression (Z-score) of each gene in each cell. The data in **(B)** are pooled from two independent experiments with n = 3-5 mice per group. Statistical analysis in **(B)** was done using one-way ANOVA combined with Tukey’s multiple comparison test. * p < 0.05, *** p < 0.001, n.s., not significant. For sorting of CD4^+^ T cells for RNA-seq analysis, two mice were infected and cured as described in **(A)**. 8 weeks post-cure the mice were killed for single cell sorting.

Building on our previous work, here we assessed the persistence of memory Th2 cells in lymphoid and non-lymphoid organs up to 3 months (12 weeks) following the cure of a primary nematode infection (**Figure 1A**). We could show that *H. polygyrus*-cured mice harbor overall stable frequencies (**Figures 1B**, **S1A**) and absolute numbers (data not shown) of memory Th2 cells up to 3 months post-cure in the PEC and siLP at 8 and 12 weeks post-cure, and a trend for mildly elevated frequencies of memory Th2 cells in the mLN and lungs of mice by 12 weeks post-cure, possibly as a contribution of recirculating populations of memory Th2 cells at these tissue sites.

To characterize the transcriptional profiles of nematode-induced memory T cells, next we performed single cell transcriptomics on sorted CD4^+^CD45.2^-^ T cells from two *H. polygyrus*-cured mice at 8 weeks post-cure (**Figures 1C–E** and **S1B**, **S2**; **Tables S1** **–** **3**). CD4^+^ T cells sorted from PEC, siLP, mLN and lung at a purity of > 90% and displayed distinct clustering primarily based on their tissue origin (**Figures 1C, D** and **S1B**). Expectedly, mLN-sorted cells (cluster I) clustered more distinctly from CD4^+^ T cells sorted from non-lymphoid organs (cluster II-V), while clusters comprising high proportions of cells sorted from the same organ (e.g. clusters IV and V) related more closely to each other. Interestingly, for clusters II and III comprising predominantly PEC and siLP-sorted cells, respectively, we observed a shared transcriptional profile of peritoneal-resident and gut-resident CD4^+^ T cells (**Figure 1D**). We also found upregulated expression of several genes like *Ccr7* (CCR7) and *Sell* (CD62-L) in mLN cells, markers typically associated with recirculating T_CM_ cells (**Figure 1E**; **Table S1**). In contrast, Th2-associated genes like *Gata3, Il4, Il5, Il13*, costimulatory markers *Icos*, *Tnfrsf4* (Ox40), *Tnfrsf9* (CD137) and *Tnfrsf18* (GITR), as well as genes like *Cd69* and *CD40l* encoding tissue-homing and activation markers were differentially expressed in PEC, siLP and lung, consistent with the expected tissue-resident phenotype of memory cells in these tissues (**Figure 1E** and **Table S1**).

To better evaluate the heterogeneous transcriptional profile of peritoneal T cells in nematode-cured mice, we further analyzed the three smaller sub-clusters of peritoneal sorted CD4^+^ T cells (**Figures 1C–E**, **S1B**, **S2**). Here, marker genes of cluster 1 (PEC1) showed prominent over-representation in gene ontology (GO) terms like *cell migration*, *cell-cell adhesion*, *interleukin-4 secretion* and *CD4-positive alpha-beta T cell differentiation*, but no *cellular response to interleukin-7*, in line with the expected transcriptional profile of quiescent memory T cells. In contrast, over-representation of genes in cluster 3 (PEC3) can be found in GO terms like *positive regulation of T cell mediated cytotoxicity*, *positive regulation of T-cell tolerance induction* and *regulation of T-cell anergy*, rather indicative of a cluster of peritoneal regulatory T cells (Treg) (**Figure S2**). Thus, a diverse pool of memory Th2 cells could be identified in the peritoneal cavity 8 weeks after cure of infection.

Overall, our RNAseq analysis in *H. polygyrus*-cured mice revealed that, at 8 weeks following cure of a primary nematode infection, mice harbor both a circulating pool of central memory-type CD4^+^ cells in secondary lymph organs like the mLN, and CD4^+^ T cells with a tissue-resident, antigen-experienced Th2 phenotype. Moreover, distinct sub-clustering of PEC cells further highlights the presence of both memory Th2 and Treg-like populations, highlighting the accumulation of diverse CD4^+^ populations in the peritoneal cavity of nematode-cured mice.

### Re-Infected Mice Display a Strong Th2 Recall Response to *H. polygyrus*

**Figure 2:**
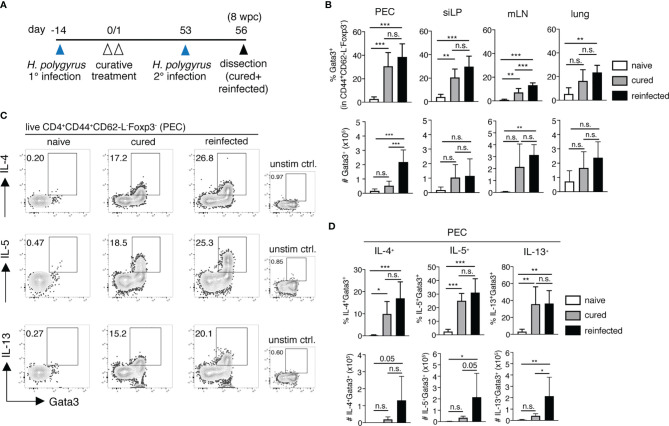
The peritoneum harbours significant memory Th2 cell expansion and cytokine release following *H. polygyrus* challenge infection. **(A)** Experimental set-up. Briefly, mice were infected *via* oral inoculation with 225 infective L3 *H. polygyrus* larvae and were cured of infection 14 days later *via* oral administration of 2mg/mouse pyrantel pamoate anti-helminthic on two consecutive days. All the cured mice were then allowed to rest. 8 weeks post-cure (day 53), a number of cured mice received a challenge *H. polygyrus* infection (reinfected group) and were sacrificed 3 days post-challenge (day 56) alongside un-challenged cured mice (cured group) and naïve controls for further analysis. **(B)** Frequencies (top panel) and absolute cell numbers (bottom panel) of Gata3^+^CD4^+^CD44^+^CD62-L^-^Foxp3^-^ memory Th2 cells in the peritoneal cavity (PEC), small intestinal lamina propria (siLP), mesenteric lymph nodes (mLN) and lungs of naïve, cured and reinfected mice. **(C)** Representative FACS plots showing cytokine^+^ PEC-resident memory Th2 cells following *in vitro* PMA/Ionomycin stimulation and corresponding unstimulated controls. **(D)** Frequencies (top panel) and absolute cell numbers (bottom panel) of IL-4^+^, IL-5^+^ and IL-13^+^ memory Th2 cells in the PEC of naïve, cured and reinfected mice. The data are pooled from two independent experiments with n = 3-5 mice per group. Statistical analysis was done using one-way ANOVA combined with Tukey’s multiple comparison test. * p < 0.05, ** p < 0.01, *** p < 0.001. n.s., not significant.

To better characterize the early memory Th2 recall responses to a secondary nematode infection, we infected and cured C57BL/6 mice of *H. polygyrus* (cured). 8 weeks post-cure, 8-12 of the cured mice were challenged with a secondary *H. polygyrus* infection and were analyzed 3 days post-challenge (reinfected, **Figure 2A**). One major novel finding here was the significant expansion of Gata3^+^CD44^+^CD62-L^-^Foxp3^-^ memory Th2 cells observed in the peritoneal compartment only, but not in the siLP, mLN or lungs of reinfected mice (**Figure 2B**). Furthermore, the expanding population of peritoneal memory Th2 cells was strongly cytokine competent, evidenced by the significantly elevated numbers of IL-4, IL-5 and IL-13-producing memory Th2 cells detected in the PEC (**Figures 2C, D**). In contrast, a significant expansion of cytokine^+^ memory Th2 cells was not observed in the siLP, mLN or lung of reinfected mice 3 days post-challenge infection (**Figures 2B, D** and **S3B–D**). Interestingly, the marked increase in peritoneal IL-5^+^ Th2 cell numbers in the *H. polygyrus*-reinfected group was further paralleled by a significant influx of eosinophils into the peritoneal cavity, but not in other tissue sites, indicative of memory Th2-driven peritoneal eosinophilia (**Figure S4**). This finding was further emphasized by a lack of notable eosinophil infiltration into the peritoneal cavity of mice at 3 days post-primary infection (data not shown) and therefore confirms peritoneal eosinophilia as a particular feature of host recall responses to a secondary nematode infection. Thus, our results reveal the presence of a prominent Th2 recall response, localized specifically in the peritoneal cavity early following secondary *H. polygyrus* infection.

### The Peritoneal Cavity Harbors Activated Parasite-Specific Ox40^+^ Memory Th2 Cells

**Figure 3:**
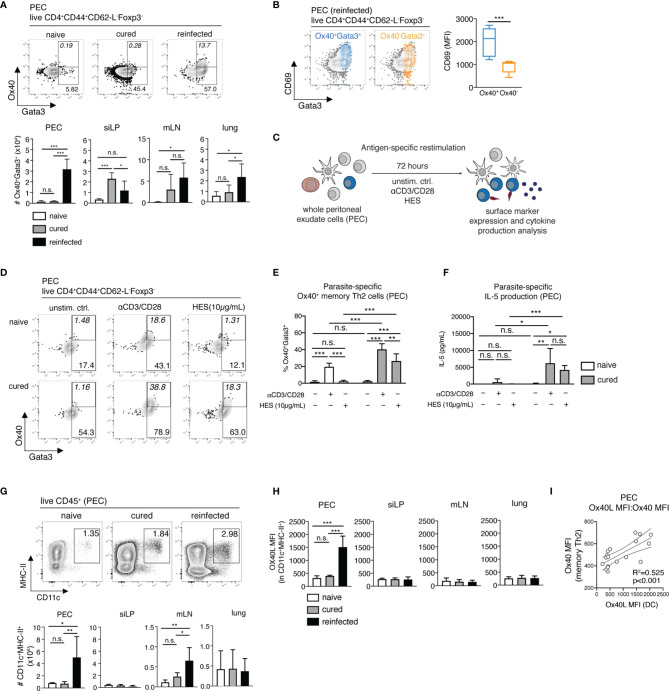
Parasite-specific Ox40^+^ memory Th2 cells and Ox40L^+^ DCs accumulate in the peritoneum during early recall responses to *H. polygyrus*. **(A)** Exemplary contour plots of Ox40 and Gata3 expression and absolute cell numbers of Ox40^+^Gata3^+^ memory Th2 cells in the PEC, siLP, mLN and lung of naïve, cured and reinfected mice. **(B)** Exemplary overlay plots and mean fluorescence intensity of CD69 expression by Ox40^+^Gata3^+^ (blue) and Ox40^-^Gata3^+^ (yellow) memory Th2 cells in the PEC of reinfected mice. **(C)** Schematic representation of the antigen-specific *in vitro* restimulation of whole PEC and mLN cells. **(D)** Exemplary plots of Ox40 and Gata3 expression by CD4^+^CD44^+^CD62-L^-^Foxp3^-^ memory Th2 cells following a 72-hour *in vitro* restimulation with αCD3/CD28 monoclonal antibodies and *H. polygyrus* excretory/secretory antigen (HES) of whole PEC cells from naïve and cured mice. **(E)** Frequencies of Ox40^+^Gata3^+^ memory Th2 cells following antigen-specific *in vitro* restimulation. **(F)** IL-5 cytokine levels in cell culture supernatants following the *in vitro* restimulation of whole PEC cells. **(G)** Exemplary FACS plots and absolute cell numbers of peritoneal CD11c^+^MHC-II^+^CD45^+^ dendritic cells (DC) in a naïve, cured and reinfected mouse. **(H)** Mean fluorescence intensities (MFI) of Ox40L on DCs in PEC, siLP, mLN and lung of naïve, cured and reinfected mice. **(I)** Correlation analysis of Ox40L expression on PEC-resident DCs and Ox40 expression on PEC-resident memory Th2 cells. Statistical analysis in **(A-C)** was done using one-way ANOVA combined with Tukey’s multiple comparison test. Statistical analysis in **(I)** was performed using Pearson’s r test. * p < 0.05, ** p < 0.01, *** p < 0.001, n.s., not significant.

Among the several upregulated costimulatory markers highlighted by the scRNA-seq analysis of non-lymphoid organs, including the PEC, was Ox40 (CD134) (**Figure 1E** and **Table S1**). Ox40 is an early inducible costimulatory molecule, normally promoting the survival and function of memory Th2 cells. Mice deficient for its only known ligand Ox40L (Ox40L^-/-^ mice) show impaired adult worm expulsion, lower IL-4 production and parasite-specific IgE responses following a secondary *H. polygyrus* infection, highlighting Ox40-Ox40L interactions as key for host immunity to a challenge nematode infection (17). Here, we profiled the *ex vivo* Ox40 expression on memory Th2 cells, comparing our cured and reinfected groups as a measure of the early induction and reactivation of host T cell responses. We found that *H. polygyrus* induces a prominent expansion of Ox40^+^ memory Th2 cell numbers specifically in the PEC, but not in other organs, as early as 3 days post-challenge infection (**Figure 3A**). Furthermore, peritoneal Ox40^+^ memory Th2 cells expressed significantly higher levels of the activation marker CD69 compared with Ox40^-^ memory Th2 cells, in line with the early inducible nature of Ox40 upon T-cell engagement with antigen-presenting cells (APCs) (**Figure 3B**).

Next, we asked whether this early memory Th2 cell reactivation in the peritoneal cavity constitutes a parasite-specific recall response. For this, we restimulated whole PEC and mLN cells from naïve and cured mice *in vitro* with anti-CD3/CD28 antibodies or with *H. polygyrus* excretory-secretory products (HES, 10µg/mL) for 72h (3 days), matching the *ex vivo* end-point of secondary infection in the reinfected group (**Figures 3C–F** and **S5**). While HES failed to induce a detectable parasite-specific Ox40 expression in naïve mice, we observed a significant increase in Ox40 expression on PEC, but not mLN cells from cured mice (**Figures 3E** and **S5A**). This was paralleled by increased levels of HES-specific IL-5 release in restimulated samples from the PEC of cured mice (**Figure 3F**). In contrast, no significant parasite-specific IL-5 was detected in restimulated mLN cells (**Figure S5B**). These findings therefore clearly indicate that the murine peritoneal compartment specifically conditions an early parasite-specific memory Th2 recall response against an intestinal parasitic nematode.

### Early Peritoneal Recall Responses to *H. polygyrus* Involve Ox40/Ox40L Interactions

To establish whether a corresponding increase in Ox40L expression on peritoneal APCs occurs during early recall responses to *H. polygyrus*, next we assessed the expression of Ox40L on CD11c^+^MHC-II^+^ dendritic cells (DCs) in the PEC, siLP, mLN and lungs of naïve, cured and reinfected mice (**Figures 3G, H**). DCs are a known source of Ox40L and provide key signaling for the priming and induction of both primary and memory Th2 cell responses (17, 18). Here, analysis of total CD11c^+^MHC-II^+^ DC numbers revealed a significant influx of DCs in the peritoneal compartment and to a lesser extent into the mLN of *H. polygyrus*-reinfected mice (**Figure 3G**). More importantly, however, a significant increase in Ox40L expression was only detectable on peritoneal DCs following secondary nematode infection (**Figure 3H**). A correlation analysis also revealed that the upregulated Ox40L expression on peritoneal DCs strongly correlates with the increased Ox40 expression on peritoneal memory Th2 cells (**Figure 3I**). Overall, these results therefore suggest that the murine peritoneal cavity is an early site of Ox40-Ox40L interactions between DCs and resident memory Th2 cells as early as 3 days post-challenge infection with *H. polygyrus*.

### The Peritoneum Downregulates VCAM-1 and ICAM-1 Expression in Response to *H. polygyrus*

**Figure 4:**
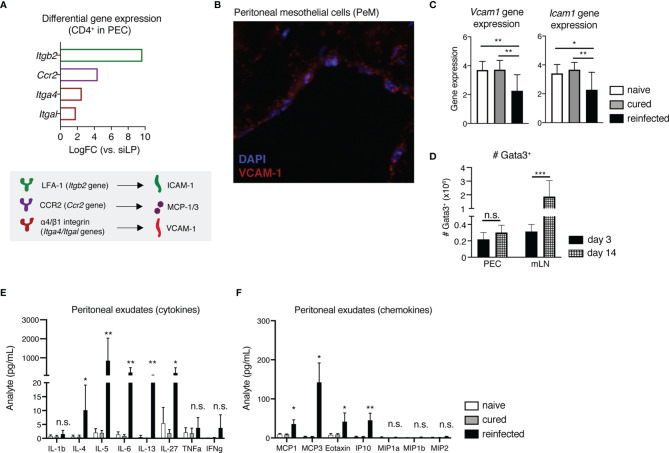
Peritoneal mesothelial cells (PeM) express several cell adhesion markers consistent with the influx of immune cells during early recall responses to *H. polygyrus* into the peritoneum. **(A)** Differential gene expression levels of *Itgb2, Ccr2, Itga4* and *Itgal* in sorted CD4^+^ T cells from the PEC in comparison with siLP-sorted cells, as quantified *via* scRNAseq (top) and a graphical legend of cell adhesion marker-ligand binding pairs (bottom). **(B)** Immunofluorescence staining of a peritoneal membrane snip from a naïve C57BL/6 mouse. DAPI nuclear staining is shown in blue and VCAM-1 staining in red. **(C)** Relative gene expression of *Vcam1* and *Icam1* in peritoneal membrane snips from naïve, cured and reinfected mice, against GAPDH as a housekeeping gene. **(D)** Absolute cell numbers of Gata3^+^CD4^+^CD44^+^CD62-L^-^Foxp3^-^ Th2 cells in the PEC and mLN of *H. polygyrus*-reinfected mice at 3 and 14 days post-challenge infection. Quantification of secreted **(E)** cytokines and **(F)** chemokines in peritoneal exudates from naïve, cured and reinfected mice. The data are pooled from two to three independent experiments with n = 2-4 mice per group. Statistical analysis was done using one-way ANOVA combined with Tukey’s multiple comparison test. * p < 0.05, ** p < 0.01, *** p < 0.001. n.s., not significant.

Considering the notable peritoneal localization of early host recall responses to secondary *H. polygyrus* infection shown here, next we aimed to decipher what mechanisms potentially drive this influx of immune cells into the peritoneal cavity of nematode-reinfected mice. The peritoneal membrane is typically lined by a layer of squamous peritoneal mesothelial (PeM) cells, expressing various cell adhesion and migration markers (19–21). Returning to our scRNAseq analysis, we found that compared with sorted gut CD4^+^ T cells, peritoneal T cells show elevated gene expression levels of cell adhesion-associated markers like *Itgb2* (encodes lymphocyte function associated antigen-1 (LFA-1), which binds induced cell adhesion marker 1 (ICAM-1)), *Ccr2* (encodes chemokine receptor 2 (CCR2), which binds monocyte chemoattractant proteins 1 and 3 (MCP-1/3)), *Itga4* and *Itgal* (encode the integrin α4β1 (CD49d/CD29), which binds vascular cell adhesion marker 1 (VCAM-1)) (**Figure 4A**).

Assessing the gene expression of *Vcam1* and *Icam1* on PeM cells *via* immunofluorescence microscopy and qPCR, we found that while in naive and cured mice there are stable gene expression levels of both *Vcam1* and *Icam1*, reinfected mice display significantly lower expression of both genes (**Figures 4B, C**). In contrast, a similar downregulation of *Vcam1* or *Icam1* gene expression was not observed in mice killed at 3 days post-primary infection (data not shown). Next, to see whether these changes in *Vcam1* and *Icam1* expression potentially correlate with later shifts in host Th2 recall responses towards the site of infection in the gut, we also assessed the numbers of Th2 cells in both the PEC and the gut-draining mLN of mice at 14 days post-challenge infection (**Figure 4D**). Here, by day 14 post-challenge we found that while total numbers of Gata3^+^ Th2 cells in the PEC were no longer increasing, there was a contrasting significant increase in Th2 cell numbers in the mLN (**Figure 4D**). In summary, these findings indicate that while the host peritoneum maintains stable levels of cell adhesion marker expression of VCAM-1 and ICAM-1 under homeostasis and following cure of a primary infection, a *H. polygyrus* challenge induces a significant downregulation of gene expression levels of both markers, likely enabling immune cell influx out of the peritoneal compartment. In addition, the peritoneal cavity appears to prime and support only early host recall responses to a strictly intestinal pathogen, as by day 14 post-challenge we start observing stronger host Th2 responses in the gut-draining lymph nodes instead.

Considering the high gene expression levels of cell adhesion and chemokine receptors LFA-1, α4β1 and CCR2 found on peritoneal compared with intestinal CD4^+^ T cells (**Figure 4A**), we also quantified the amounts of secreted cytokines and chemokines in peritoneal exudates of naïve, cured and reinfected mice using a magnetic bead multiplex assay (**Figure 4E**). We detected expectedly elevated levels of Th2 cytokines like IL-4, IL-5, IL-6 and IL-13, in line with the marked reactivation and expansion of peritoneal memory Th2 cells shown earlier. One novel finding here was the markedly elevated levels of the APC-secreted immunomodulatory cytokine IL-27 in the peritoneal cavity of reinfected mice, fitting well with the observed influx of peritoneal DCs following *H. polygyrus* challenge (**Figure 4E**). Considering the known immunomodulatory functions of both IL-27 and nematode-derived molecules, we also checked whether HES/IL-27-mediated signaling potentially contributes to the observed downregulation of *Vcam1* and *Icam1* in the peritoneum of reinfected mice. For this, we performed an *in vitro* restimulation of whole peritoneal membrane snips with either the pro-inflammatory cytokine IFNγ, known to increase ICAM-1 and VCAM-1 expression on human pleural mesothelial cells (22), or with HES either in the presence or absence of IL-27. Here, while IFNγ restimulation induced an increase in gene expression of *Icam1*, but not *Vcam1*, HES and IL-27 did not significantly modulate neither *Vcam1* nor *Icam1* expression on murine PeM cells *in vitro*, indicating that alternative signaling mechanisms in the peritoneum regulate cell adhesion marker expression in response to a nematode infection (**Figure S6**). Finally, we found that reinfected mice displayed significantly elevated levels of several chemokines. including MCP-1/3 and the eosinophil-attracting chemokine Eotaxin (CCL11) (**Figure 4F**), fitting with the differential gene expression of CCR2 on peritoneal T cells and the marked peritoneal eosinophilia, respectively (**Figure 4F**).

In summary, we could show that within the murine peritoneal cavity, the peritoneal membrane lined by PeM cells expresses stable levels of cell adhesion markers VCAM-1 and ICAM-1 under steady state and following cure of a primary infection, and significantly downregulates the gene expression of both markers in response to a challenge nematode infection. In line with this finding, within our scRNA-seq data we also observe high differential gene expression of the binding molecules for both VCAM-1 (α4β1) and ICAM-1 (LFA-1) on peritoneal CD4^+^ T cells, therefore indicating that the notable recruitment and retention of memory Th2 cells in the peritoneal cavity of mice cured of a primary *H. polygyrus* infection is potentially cell adhesion marker-driven. Finally, we highlight the presence of significantly elevated levels of several APC-derived and signature Th2 cytokines, as well as chemokines associated with monocyte recruitment and activation in peritoneal exudates of reinfected mice, further highlighting the murine peritoneal compartment as a site of diverse immune cell recruitment, retention and reactivation in response to an otherwise strictly enteric pathogen.

## Discussion

The formation, distribution and rapid recall abilities of memory T cells are crucial elements of immunological memory responses. Here, we focused on characterizing the early Th2 recall responses to a challenge infection with the small intestinal nematode *H. polygyrus* in mice. We could show that along with the siLP and the lung, the peritoneal cavity harbors a pool of CD4^+^ T cells with a typical tissue-resident memory transcriptional profile, including upregulated expression of costimulatory markers like Ox40 and tissue retention markers like CD69 (23, 24). As an early inducible marker, Ox40 normally promotes the survival and function of memory Th2 cells *via* positive regulation of anti-apoptotic proteins like Bcl-2 and Bcl-xl (25–27). Several studies have shown that Ox40/Ox40L signaling is critical for memory Th2 cell functionality during allergic airway inflammation in mice (27–29). For nematode infections, Ox40L^-/-^ mice have been shown to exhibit impaired adult worm expulsion, and weaker IL-4 and IgE responses to secondary *H. polygyrus* infection, confirming Ox40/Ox40L interactions as important for efficient host recall responses to intestinal parasitic nematodes (17). Here, we highlight for the first time the host peritoneal cavity as a key site of parasite-specific upregulation of Ox40 expression on memory Th2 cells, paralleled by an influx of peritoneal Ox40L^+^ DCs as early as 3 days following a secondary infection with an otherwise strictly enteric nematode. Moreover, even though nematode infections are typically associated with eosinophil infiltration at the site of infection, we now demonstrate that a secondary *H. polygyrus* infection also induces a significant early eosinophil accumulation at distal sites such as the peritoneal cavity. This therefore further highlights a novel importance of the peritoneal compartment for priming and harboring early recall responses to infections with gut-restricted pathogens.

Under homeostasis, during infection or inflammation the cellular composition of the peritoneal cavity is typically regulated *via* the bidirectional migration of immune cells, a process reliant on chemokine receptors and cell adhesion markers (30–33). In our previous study, we found that the majority of memory Th2 cells from the peritoneal cavity of nematode-cured mice express the α4 integrin subunit (CD49d), while less than 10% of intestinal memory Th2 cells were CD49d^+^ (4). Importantly, *via* interactions with their complementary receptors α4β1 and LFA-1, the cell adhesion markers VCAM-1 and ICAM-1 have been shown to mediate mononuclear leukocyte infiltration, increase T cell and APC avidity and thus to modulate T cell activation and differentiation (34, 35). In line with previous findings (20) and expanding on our own work (4), here we can now show that the murine peritoneal membrane, lined by PeM cells, is a source of both VCAM-1 and ICAM-1 under homeostasis and following cure of a primary *H. polygyrus* infection, thus explaining the prominent recruitment and retention of tissue-resident CD49d^+^ memory Th2 cells in the peritoneal compartment, rather than the gut of nematode-cured mice.

Several studies have previously shown that human pleural mesothelial cells upregulate their ICAM-1 expression and serve as chemokine sources in response to bacterial infection, thus directly participating in the recruitment of monocytes and CD4^+^ T cells (22, 36, 37). In contrast, blocking ICAM-1 leads to lower CD4^+^ T cell activation *in vitro* (22) and to a significant reduction in monocyte transmigration in response to chemotactic stimuli (36). In light of this data, an additional new finding in our current study is that the murine peritoneum can directly respond to a challenge intestinal nematode infection by rather decreasing its gene expression levels of *Vcam1* and *Icam1* early during recall responses to *H. polygyrus*, likely enabling the immune cell efflux out of the peritoneal cavity. Furthermore, considering the initial expansion of peritoneal Th2 cells during early recall responses at 3 days post-challenge in the PEC only, and the contrasting later expansion of Gata3^+^ cells in the gut-draining lymph nodes by 14 days post-challenge infection, our findings therefore suggest that the host peritoneal compartment participates in and supports specifically early recall responses to the enteric parasite *H. polygyrus*. Considering the gut-restricted colonization of *H. polygyrus*, efficient control of this pathogen ultimately relies on strong host Th2 responses in the siLP and mLN, rather than in peripheral sites like the peritoneal cavity. The efflux of immune cell subsets like B cells out of the peritoneum requires the downregulation of integrin molecules, correlating with a corresponding increasing appearance of B cells in lymph nodes (33). Even though here we don’t show evidence of lower integrin expression on peritoneal-resident Th2 cells following *H. polygyrus* reinfection, we rather present evidence of significantly downregulated *Vcam1* and *Icam1* gene expression levels in the peritoneum of nematode-reinfected mice at 3 days post-challenge, coupled with increasing numbers of Th2 cells in the mLN by 14 days post-secondary infection. Our findings therefore support one potential new mechanism of immune cell recirculation *via* the peritoneum, where nematode-induced LFA-1 and CD49d-expressing CD4^+^ T cells take up residence in the host peritoneal cavity, attracted by the stable expression levels of ICAM-1 and VCAM-1 following the cure of a primary *H. polygyrus* infection. Upon nematode reinfection, the peritoneal membrane starts downregulating its cell adhesion marker expression to allow for immune cell efflux out of the peritoneum and towards the site of infection in the gut, complementing the already initiated *de novo* induction of Th2 effector cells expected in the mLN of reinfected mice.

Despite the upregulated levels of secreted chemokines like MCP-1/3 and Eotaxin in peritoneal exudates, we did not observe a corresponding elevated gene expression of either marker in the peritoneal membrane of reinfected mice 3 days post-challenge (data not shown). This therefore indicates that other immune cell subsets such as peritoneal macrophages, rather than PeM cells, serve as important chemokine sources, potentially complementing APC-driven immune cell reactivation.

Several studies in recent years have highlighted the APC-derived IL-27 as an immunomodulatory cytokine, capable of regulating Th1 cell differentiation (38), chemokine production in human bronchial epithelial cells (39), as well as mediating intestinal epithelial barrier function (40). IL-27 also appears to inhibit activation-induced cell death (AICD) of CD4^+^ T cells, sustaining their expansion upon activation (41). Similarly, IL-27 is reported to enhance eosinophil survival, migration and activation (42). The high concentrations of IL-27 in the peritoneal exudates are therefore in line with the strong expansion and parasite-specific reactivation of peritoneal Th2 cells, as well as with the prominent peritoneal eosinophil influx in *H. polygyrus*-reinfected mice and points to a role for IL-27 in T cell and granulocyte attraction and activation in the host peritoneal cavity during early recall responses to infection. Finally, *H. polygyrus* itself is also a potent immunomodulator and can regulate host immune responses *via* the release of a diverse cocktail of parasite-derived molecules (43–45). In the current study, we observed a significant increase of both DCs and secreted IL-27 levels in the peritoneal cavity of reinfected mice, but neither *H. polygyrus*-released products (HES), nor IL-27 induced a decrease in the cell adhesion markers VCAM-1 and ICAM-1 *in vitro*, possibly as a result of a lack of IL-27 receptor expression on murine PeM cell.

In summary, our study places a novel focus on the involvement of the host peritoneal cavity in driving the recruitment and reactivation of memory Th2 cells, DCs and eosinophils during early recall responses to a gut-restricted parasitic nematode. We could show that the peritoneal compartment of nematode-reinfected mice harbors notable numbers of parasite-specific, functional memory Th2 cells, as well as a significant influx of both DCs and eosinophils. Furthermore, we show for the first time that the murine peritoneum, typically lined by PeM cells, responds to an intestinal nematode infection by decreasing its gene expression of the cell adhesion markers VCAM-1 and ICAM-1 as early as 3 days post-challenge infection, despite otherwise stable expression of both markers following cure of a primary infection. We therefore highlight the host peritoneal compartment as an important player in the development of early Th2 recall immune responses to challenge infections with an enteric pathogen.

## Materials and Methods

### Mice and Nematode Infection

Wild-type female C57BL/6 mice (age 8-10 weeks) were purchased from Janvier Labs (Saint-Berthevin, France). All animals were maintained under specific pathogen-free (SPF) conditions and were fed standard chow *ad libidum*. *H. polygyrus* was maintained by serial passage in C57BL/6 mice (H0099/13). Mice were infected with 225 third-stage infective (L3) *H. polygyrus* larvae *via* oral gavage in 200µL drinking water. For cure of infection, mice were treated on two consecutive days during the acute stage of infection (day 14-15) with 2mg pyrantel pamoate (Sigma, St. Louis, MO, USA) in 200µL drinking water *via* oral gavage. On indicated days, mice were sedated *via* isofluorane inhalation, followed by cervical dislocation. The efficacy of curative treatment with pyrantel pamoate was confirmed at dissection by the lack of adult worms in the small intestine of cured mice. All animal experiments were performed in accordance with the National Animal Protection Guidelines and approved by the German Animal Ethics Committee for the Protection of Animals (LAGeSo, G0176/20).

### Preparation of Single Cell Suspensions

The isolation of PEC, siLP and mLN was performed as previously described (4). For the isolation of lung cells, the chest cavity was opened *via* incision and the whole lung was perfused with 20mL ice-cold PBS *via* puncture to the heart using a 27G needle and syringe. Each lung was then removed and manually minced into small pieces. The minced tissue was then transferred to a 50mL falcon tube containing 10mL digestion medium (150µg/mL collagenase D and DNase I in PBS). The samples were incubated for 1 hour at 37°C under continuous shaking at 250rpm. Following the digestion step, the lung tissue was passed through a 70µm cell strainer into a fresh 50mL falcon tube. After erythrocyte lysis, the lung samples were washed and resuspended in cRPMI. All cell suspensions were counted using a CASY automated cell counter (Roche-Innovatis, Reutlingen, Germany).

### CD4^+^ T Cell Sorting and Library Preparation

Cell suspensions from PEC, siLP, mLN and lung were obtained and counted as described above from two *H. polygyrus*-cured C57BL/6 mice at 8 weeks post-cure. Immediately prior to dissection, each mouse was injected intravenously with anti-mouse CD45.2-A700 (clone 104) to allow for the exclusion of any blood-derived cells in circulation. Each sample was stained with anti-mouse CD4-PerCP (clone RM4-5) and was sorted for live single CD4^+^CD45.2^-^ T cells on a FACS Aria cell sorter (BD Biosciences, Heidelberg, Germany).

Capture and processing of single CD4^+^ T cells was performed using the Fluidigm C1 autoprep system. Sorted CD4^+^ T cells (50 cells/µl) were mixed with (ratio 6/4) C1 Cell Suspension Reagent (Fluidigm) and subsequently loaded onto a 5-10-μm-diameter C1 Integrated Fluidic Circuit (IFC; Fluidigm From the nine chips used, we captured 517 single cells (59.8%). ERCC (External RNA Controls Consortium) spike-in RNAs (Ambion, Life Technologies) were added to the lysis mix. Reverse transcription and cDNA preamplification were performed using the SMARTer Ultra Low RNA kit (Clontech). Sequencing libraries were prepared using Nextera XT DNA Sample Preparation kit with 96 indices (Illumina), according to the protocol supplied by Fluidigm. Size distribution in final libraries was analyzed by Bioanalyzer High Sensitivity DNA assay (Agilent) and library concentrations were quantified by Qubit High Sensitivity DNA assay (ThermoFisher). A total of 384 libraries were pooled equimolar and sequenced on an Illumina HiSeq 1500 system in rapid mode v1 by a paired-end dual-indexing run with 2x 125 cycle reads. Subsequently, the generation and trimming of fastq files was performed followed by alignment of the reads to mm10 mouse genome version.

Quality control of the raw fastq files was done with FastQC (https://www.bioinformatics.babraham.ac.uk/projects/fastqc/). Trimming of the raw reads was done with Trimmomatic v0.36, with the following filters and settings in this order: Nextera adaptors, ILLUMINACLIP:${ADAPTER}:2:30:10 (adapter trimming), LEADING:3 (5’ Trimming phred<3), TRAILING:3 (3’ Trimming phred<3), SLIDINGWINDOW:3:20 (mean phred score in sliding window size 3 had to be at least 20), MINLEN:36 (discard reads shorter than 36bp) (46).

### RNA Sequencing and Data Analysis

Single cell RNA-Seq data were analyzed using R and the indicated R-packages. At first, counts per gene were calculated from the bam files by adding all counts mapped to the region of a corresponding gene (Rsamtools) (47). Quality control of the count data was performed with the R-package scater (48, 49). Cells with low library size, low numbers of features or a high number of mitochondrial sequences were filtered out using the quickPerCellQC function. Features (genes) without any count in all remaining cells were removed from the dataset and count data were normalized and log2-transformed after adding a pseudocount to each value. Variances in the expression profile of each gene were modelled based on a fitted mean-variance trend (scran - modelGeneVar) (50), decomposing it into technical and biological components. Hypervariable expressed genes were identified by applying a threshold of 4 for the variances of the biological component. 500 genes with the highest biological variances were selected for dimension reduction using the UMAP algorithm (scater) (48). In order to further stratify the dataset to genes driving systemic substructures and to remove noise, pairwise correlations between all genes were calculated (scran - correlatePairs) (50). P-values were adjusted for multiple testing by calculating the false discovery rates and significant genes were selected by a fdr below 0.05. These genes were used in a hierarchical clustering with euclidean distances and ward.D2 linkage (R - inbuilt dist and hclust function) (49). Five clusters were defined by the cutTreeDynamic function (dynamicTreeCut) (51). The dendrogram information (order of the columns), as well as the cluster definitions were used in the heatmap representation of pre-selected genes (heatmap3) (52).

Differential expression of genes between PEC and other tissues were determined by fitting negative binomial generalized linear models combined with likelihood ratio tests (edgeR - glmFIT and glmLRT) (53). Ranks were determined for each comparison and the whole set was ordered according to the minimal rank of each gene.

Marker genes for the three PEC clusters were determined by combined pairwise comparisons (t-Tests) using the findMarkers function of the scran package. Significant genes were selected by a combined p-value below 0.05. Functional annotation and overrepresentation analysis of these gene sets was done by DAVID using the R package “RDAVIDWebService” (54). Comparison of functional aspects between the three PEC clusters was done using the R-package “clusterProfiler” (55). Results for the biological branch of the gene ontology system were further processed. GO terms with overrepresentation in all PEC clusters were removed to focus for the differences. Each term was mapped (if possible) to the significant most specific (child) term. 15 top-ranking terms from this processed set for each PEC cluster were then compared in a dotplot.

### Flow Cytometry

The antibodies used for the detection of surface and intracellular markers are described in **Table S4**. Dead cells were excluded using eFluor780 or eF560 fixable viability dye (Thermo Fisher, Waltham, USA). For intracellular staining of cytokines and transcription factors, cells were fixed and permeabilized using the Fixation/Permeabilization kit and Permeabilization buffer from ThermoFisher/eBioscience. Samples were analyzed on a Canto II flow cytometer and on an Aria cell sorter (BD Biosciences, Heidelberg, Germany). The data was analyzed using FlowJo software Version 10 (Tree star Inc., Ashland, OR, USA). The data for naïve, cured (8wpc and 12wpc) and reinfected mice presented in this study are pooled from two independent experiments with 3-5 mice per group in each experiment.

### Cell Culture and *In Vitro* Re-Stimulation

For the analysis of parasite-specific Ox40 expression and IL-5 secretion, 5x10^5^ whole PEC or mLN cells were plated out per well in a round-bottom 96-well cell culture plate in a final volume of 200µL RPMI medium, containing 10% FCS, 100U/mL penicillin and 100µg/mL streptomycin (all from PAA, Pasching, Austria). The cells were stimulated with either anti-CD3/CD28 antibodies (1µg/mL) or *H. polygyrus* excretory-secretory (HES) antigen (10µg/mL). The cells were then incubated for 72 hours at 37°C and 5% CO_2_. After 72 hours, the cell pellets were collected and stained for flow cytometric analysis, as described above, while the culture supernatants were collected and used for quantifying IL-5 secretion *via* ELISA, as described below. The data presented for this assay are pooled from two independent experiments with 2-4 mice per group in each experiment.

### Quantification of Parasite-Specific IL-5 Production *In Vitro*

Parasite-specific IL-5 production of restimulated PEC and mLN cells was measured *via* sandwich ELISA using the Mouse IL-5 Uncoated ELISA kit as per the manufacturer’s instructions (Invitrogen). Absorbance was measured on a Biotek Synergy H1 Hybrid Reader at a 450nm wavelength.

### Eosinophil Peroxidase Quantification in Serum

Eosinophil peroxidase (EPO) levels in blood serum were quantified using the Mouse Eosinophil Peroxidase (EPX) ELISA Kit as per the manufacturer’s instructions (DLdevelop, Jiangsu, PRC). Serum samples were diluted 1:5 in Diluent Buffer and were added to the plate with serially diluted standards. Absorbance was measured on a Biotek Synergy H1 Hybrid Reader at a 450nm wavelength. The data presented for this assay are pooled from two independent experiments with 3-5 mice per group in each experiment.

### Immunohistochemistry

Sections were cut from formalin-fixed and paraffin-embedded tissues, dewaxed and subjected to a heat-induced epitope-retrieval step. For immunofluorescence, endogenous peroxidase was blocked (Dako REAL Peroxidase-Blocking Solution, Agilent, Santa Clara, CA, U.S.) and sections were incubated with anti-VCAM-1 (clone EPR5047, Abcam, Cambridge, U.K.). For detection, the EnVision+ Single Reagent (HRP. Rabbit, Agilent) and the Opal 570 reagent (Akoya Biosciences, MA, U.S.) were used. Nuclei were stained with DAPI (Sigma-Aldrich Chemie GmbH, Munich, Germany) and slides were cover-slipped in Fluoromount G (Southern Biotech, Birmingham, AL, U.S.). Stained sections were analyzed in a blinded manner using an AxioImager Z1 microscope (Carl Zeiss Microscopy Deutschland GmbH, Oberkochen, Germany). The immunohistochemistry data shown here are representative of two independent experiments with 2-4 mice per group in each experiment.

### Quantitative Real-Time PCR (qPCR)

At necropsy, 1cm^2^ tissue snips of peritoneal membrane were excised, flash-frozen in liquid nitrogen and were stored at -80°C. RNA was isolated using the Monarch Total RNA Miniprep kit (New England BioLabs, MA, USA) according to the manufacturer’s instructions. 2μg of RNA was then reverse-transcribed to cDNA using the High-Capacity RNA-to-cDNA kit (Applied Biosystems, Foster City, CA, USA). Relative gene expression was determined *via* quantitative real-time PCR (qPCR) using 10 ng of cDNA and FastStart Universal SYBR Green Master Mix (Roche). Primer pairs are described in **Table S5**. Efficiencies for each primer pair were determined by generating a standard curve. mRNA expression was normalized to the housekeeping gene glyceraldehyde 3-phosphate dehydrogenase (GAPDH) and was calculated by the Roche Light Cycler 480 software. The qPCR data are pooled from three independent experiments with 2-4 mice per group in each experiment.

### Magnetic Bead Immunoassay and Peritoneal Analyte Quantification

At necropsy, 1mL of PBS buffer was carefully injected and retracted from the cavity of naïve, cured and reinfected mice using a 20G needle and syringe. These peritoneal exudate samples were then centrifuged, the supernatants were collected and stored at -20°C for performing a multiplex sandwich ELISA-based Luminex immunoassay using the Mouse Cytokine & Chemokine 26-plex ProcartaPlex kit as per the manufacturer’s instructions. The results were read using a Luminex MagPix machine. The data presented for this assay are pooled from two independent experiments with 3-4 mice per group in each experiment.

### *In Vitro* PeM cell restimulation

For the *in vitro* restimulation of murine PeM cells, snips from naïve C57BL/6 mice were carefully excised and were plated out in 200µL cRPMI medium in 96-well plates (one snip per well). Snips from each mouse were then either left unstimulated as a negative control or were incubated with IFNγ (10ng/mL), HES (10µg/mL) or HES and IL-27 (10µg/mL + 50ng/mL, respectively) for 24h at 37°C, 5% CO_2_. After the 24h incubation, the snips were then snap frozen and stored at -80°C for later RNA extraction, reverse transcription and qPCR analysis of *Vcam1* and *Icam1* gene expression levels as described above. The data presented for this assay are pooled from two independent experiments with 2-4 mice per group in each experiment.

### Statistical Analysis

Statistical analysis of FACS data was performed using GraphPad Prism software version 9.0.1 (La Jolla, CA, USA). Results are displayed as mean ± SD and significance is displayed as *p<0.05, **p<0.01, ***p<0.001. Results were tested for normal distribution using the Shapiro-Wilk normality tests, followed by ANOVA or Kruskal-Wallis combined with Tukey’s or Dunn’s multiple comparison testing.

## Data Availability Statement

The RNAseq data presented in the study are deposited in the GEO database (NCBI) repository, accession number GSE198203.

## Ethics Statement

The animal study was reviewed and approved by LAGeSO, G0176/20.

## Author Contributions

IAY, SS, BS, and SH planned and designed the study. IAY, SS, and AAK performed experiments, data analysis and interpretation. KV and UK performed the library preparation and single-cell RNA sequencing. KJ performed the bioinformatics analysis. KJ and BS interpreted the scRNA-seq data. AAK performed and interpreted the immunohistochemistry analysis. IAY wrote the manuscript. IAY, KJ, SS, KV, UK, AAK, BS, and SH edited the manuscript. BS and SH supervised the study and provided critical review of the manuscript. All authors contributed to the article and approved the submitted version.

## Funding

This study was supported by a German Research Foundation (DFG) grant HA 2542/8-1 awarded to SH.

## Conflict of Interest

The authors declare that the research was conducted in the absence of any commercial or financial relationships that could be construed as a potential conflict of interest.

## Publisher’s Note

All claims expressed in this article are solely those of the authors and do not necessarily represent those of their affiliated organizations, or those of the publisher, the editors and the reviewers. Any product that may be evaluated in this article, or claim that may be made by its manufacturer, is not guaranteed or endorsed by the publisher.

## References

[1] Pradeu T Du P,L. Immunological Memory: What’s in a Name? Immunol Rev (2018) 283:7–20. doi: 10.1111/imr.12652 29664563

[2] Jameson SC Masopust D. Understanding Subset Diversity in T Cell Memory. Immunity (2018) 48:214–26. doi: 10.1016/j.immuni.2018.02.010 PMC5863745 29466754

[3] Nguyen QP Deng TZ Witherden DA Goldrath AW. Origins of CD4 + Circulating and Tissue-Resident Memory T-Cells. Immunology (2019) 157:3–12. doi: 10.1111/imm.13059 30897205 PMC6459775

[4] Steinfelder S Rausch S Michael D Kühl AA Hartmann S. Intestinal Helminth Infection Induces Highly Functional Resident Memory Cd4+T Cells in Mice. Eur J Immunol (2017) 47:353–63. doi: 10.1002/eji.201646575 27861815

[5] King EM Kim HT Dang NT Michael E Drake L Needham C. Immuno-Epidemiology of Ascaris Lumbricoides Infection in a High Transmission Community: Antibody Responses and Their Impact on Current and Future Infection Intensity. Parasite Immunol (2005) 27:89–96. doi: 10.1111/j.1365-3024.2005.00753.x 15882235

[6] Jia TW Melville S Utzinger J King CH Zhou XN. Soil-Transmitted Helminth Reinfection After Drug Treatment: A Systematic Review and Meta-Analysis. PloS Negl Trop Dis (2012) 6(5):e1621. doi: 10.1371/journal.pntd.0001621 22590656 PMC3348161

[7] Butterworth AE Fulford AJ Dunne DW Ouma JH Sturrock RF. Longitudinal Studies on Human Schistosomiasis. Philos Trans R Soc Lond B Biol Sci (1988) 321(1207):495–511. doi: 10.1098/rstb.1988.0105 2907155

[8] Faulkner H Turner J Kamgno J Pion SD Boussinesq M Bradley J. Age- and Infection Intensity-Dependent Cytokine and Antibody Production in Human Trichuriasis: The Importance of Ige. J Infect Dis (2002) 185:665–72. doi: 10.1086/339005 11865424

[9] Rausch S Huehn J Kirchoff D Rzepecka J Schnoeller C Pillai S. Functional Analysis of Effector and Regulatory T Cells in a Parasitic Nematode Infection. Infect Immun (2008) 76:1908–19. doi: 10.1128/IAI.01233-07 PMC2346705 18316386

[10] Rausch S Hoehn J Loddenkemper C Hepworth M Klotz C Sparwasser T. Establishment of Nematode Infection Despite Increased Th2 Responses and Immunopathology After Selective Depletion of Foxp3+ Cells. Eur J Immunol (2009) 39:3066–77. doi: 10.1002/eji.200939644 19750483

[11] Blankenhaus B Klemm U Eschbach ML Sparwasser T Hoehn J Kühl AA. Strongyloides Ratti Infection Induces Expansion of Foxp3+ Regulatory T Cells That Interfere With Immune Response and Parasite Clearance in Balb/C Mice. J Immunol (2011) 186:4295–305. doi: 10.4049/jimmunol.1001920 21335490

[12] Maizels RM McSorley HJ. Regulation of the Host Immune System by Helminth Parasites. J Allergy Clin Immunol (2016) 138:666–75. doi: 10.1016/j.jaci.2016.07.007 PMC5010150 27476889

[13] Harvie M Camberis M Tang SC Delahunt B Paul W Le Gros G. The Lung is an Important Site for Priming CD4 T-Cell-Mediated Protective Immunity Against Gastrointestinal Helminth Parasites. Infect Immun (2010) 78:3753–62. doi: 10.1128/IAI.00502-09 PMC2937440 20605978

[14] Obata-Ninomiya K Ishiwata K Nakano H Endo Y Ichikawa T Onodera A. Cxcr6+St2+ Memory T Helper 2 Cells Induced the Expression of Major Basic Protein in Eosinophils to Reduce the Fecundity of Helminth. Proc Natl Acad Sci (2018) 115:E9849–58. doi: 10.1073/pnas.1714731115 PMC6196506 30275296

[15] Filbey KJ Camberis M Chandler J Turner R Kettle AJ Eichenberger RM. Intestinal Helminth Infection Promotes IL-5- and CD4 + T Cell-Dependent Immunity in the Lung Against Migrating Parasites. Mucosal Immunol (2019) 12:352–62. doi: 10.1038/s41385-018-0102-8 30401814

[16] Mohrs K Harris DP Lund FE Mohrs M. Systemic Dissemination and Persistence of Th2 and Type 2 Cells in Response to Infection With a Strictly Enteric Nematode Parasite. J Immunol (2005) 175:5306–13. doi: 10.4049/jimmunol.175.8.5306 16210636

[17] Ekkens MJ Liu Z Whitmire J Xiao S Foster A Pesce J. The Role of Ox40 Ligand Interactions in the Development of the Th2 Response to the Gastrointestinal Nematode Parasite Heligmosomoides Polygyrus. J Immunol (2003) 170:384–93. doi: 10.4049/jimmunol.170.1.384 12496423

[18] Jenkins SJ Perona-Wright G Worsley AGF Ishii N MacDonald AS. Dendritic Cell Expression of OX40 Ligand Acts as a Costimulatory, Not Polarizing, Signal for Optimal Th2 Priming and Memory Induction *In Vivo* . J Immunol (2007) 179:3515–23. doi: 10.4049/jimmunol.179.6.3515 17785785

[19] Shimotsuma M Kawata M Hagiwara A Takahashi T. Milky Spots in the Human Greater Omentum. Macroscopic and Histological Identification. Acta Anat (1989) 136:211–6. doi: 10.1159/000146888 2603633

[20] Suassuna JHR Neves FCD Hartley RB Ogg CS Cameron JS. & Immunohistochemical Studies of the Peritoneal Membrane and Infiltrating Cells in Normal Subjects and in Patients on CAPD. Kidney Int (1994) 46:443–54. doi: 10.1038/ki.1994.292 7967356

[21] Jonjic BN Peri G Bernasconi S Sciacca FL Colotta F Pelicci P. Expression of Adhesion Molecules and Chemotactic Cytokines in Cultured Human Mesothelial Cells. J Exp Med (1992) 176:1165–74. doi: 10.1084/jem.176.4.1165 PMC2119405 1383376

[22] Yuan ML Tong ZH Guang X Zhang JC Wang XJ Ma WL. Regulation of Cd4+ T Cells by Pleural Mesothelial Cells *via* Adhesion Molecule-Dependent Mechanisms in Tuberculous Pleurisy. PloS One (2013) 8:1–10. doi: 10.1371/journal.pone.0074624 PMC3777994 24069325

[23] Carbone FR. Tissue-Resident Memory T Cells and Fixed Immune Surveillance in Nonlymphoid Organs. J Immunol (2015) 195:17–22. doi: 10.4049/jimmunol.1500515 26092813

[24] Schenkel JM Masopust D. Tissue-Resident Memory T Cells. Immunity (2014) 41:886–97. doi: 10.1016/j.immuni.2014.12.007 PMC4276131 25526304

[25] Gramaglia I Weinberg AD Lemon M Croft M. Ox-40 Ligand: A Potent Costimulatory Molecule for Sustaining Primary CD4 T Cell Responses. J Immunol (1998) 161:6510–7. 9862675

[26] Rogers PR Song J Gramaglia I Killeen N Croft M. OX40 Promotes Bcl-Xl and Bcl-2 Expression and Is Essential for Long-Term Survival of CD4 T Cells. Immunity (2001) 15:445–55. doi: 10.1016/s1074-7613(01)00191-1 11567634

[27] Salek-Ardakani S Song J Halteman BS Jember AGH Akiba H Yagita H. Ox40 (Cd134) Controls Memory T Helper 2 Cells That Drive Lung Inflammation. J Exp Med (2003) 198:315–24. doi: 10.1084/jem.20021937 PMC2194076 12860930

[28] Jember AGH Zuberi R Liu FT Croft M. Development of Allergic Inflammation in a Murine Model of Asthma Is Dependent on the Costimulatory Receptor Ox40. J Exp Med (2001) 193:387–92. doi: 10.1084/jem.193.3.387 PMC2195923 11157058

[29] Gracias DT Sethi GS Mehta AK Miki H Gupta RK Yagita H. Combination Blockade of Ox40l and Cd30l Inhibits Allergen-Driven Memory Th2 Reactivity and Lung Inflammation. J Allergy Clin Immunol (2021) 147(6):1–14. doi: 10.1016/j.jaci.2020.10.037 33160971 PMC8096862

[30] Ohnmacht C Pullner A Rooijen Nv Voehringer D. Analysis of Eosinophil Turnover *In Vivo* Reveals Their Active Recruitment to and Prolonged Survival in the Peritoneal Cavity. J Immunol (2017) 179:4766–74. doi: 10.4049/jimmunol.179.7.4766 17878375

[31] Ruiz-Campillo MT Hernandez VM Escamilla A Stevenson M Perez J Martinez-Moreno A. Immune Signatures of Pathogenesis in the Peritoneal Compartment During Early Infection of Sheep With Fasciola Hepatica. Sci Rep (2017) 7:1–14. doi: 10.1038/s41598-017-03094-0 28584245 PMC5459796

[32] Höpken UE Winter S Achtman AH Krüger K Lipp M. CCR7 Regulates Lymphocyte Egress and Recirculation Through Body Cavities. J Leukoc Biol (2010) 87:671–82. doi: 10.1189/jlb.0709505 20028772

[33] Berberich S Dähne S Schippers A Peters T Müller W Kremmer E. Differential Molecular and Anatomical Basis for B Cell Migration Into the Peritoneal Cavity and Omental Milky Spots. J Immunol (2008) 180:2196–203. doi: 10.4049/jimmunol.180.4.2196 18250426

[34] Leitner J Grabmeier-Pfistershammer K Steinberger P. Receptors and Ligands Implicated in Human T Cell Costimulatory Processes. Immunol Lett (2010) 128:89–97. doi: 10.1016/j.imlet.2009.11.009 19941899

[35] Schmidt EP Kuebler WM Lee WL Downey GP. Adhesion Molecules: Master Controllers of the Circulatory System. Compr Physiol (2016) 6:945–73. doi: 10.1002/cphy.c150020 27065171

[36] Nasreen N Mohammed KA Ward MJ Antony VB. Mycobacterium-Induced Transmesothelial Migration of Monocytes Into Pleural Space: Role of Intercellular Adhesion Molecule-1 in Tuberculous Pleurisy. J Infect Dis (1999) 180:1616–23. doi: 10.1086/315057 10515824

[37] Ye ZJ Zhou Q Yuan ML Du RH Yang WB Xiong XZ. Differentiation and Recruitment of Il-22-Producing Helper T Cells Stimulated by Pleural Mesothelial Cells in Tuberculous Pleurisy. Am J Respir Crit Care Med (2012) 185:660–9. doi: 10.1164/rccm.201107-1198OC 22199006

[38] Lucas S Ghilardi N Li J Sauvage FJDe. IL-27 Regulates IL-12 Responsiveness of Naïve CD4+ T Cells Through Stat1-Dependent and -Independent Mechanisms. Proc Natl Acad Sci USA (2003) 100:15047–52. doi: 10.1073/pnas.2536517100 PMC299900 14657353

[39] Pereira ABM de Oliveira JR Teixeira MM da Silva PR Junior VR Rogerio ADeP. IL-27 Regulates IL-4-Induced Chemokine Production in Human Bronchial Epithelial Cells. Immunobiology (2021) 226:152029. doi: 10.1016/j.imbio.2020.152029 33278712

[40] Diegelmann J Olszak T Göke B Blumberg RS Brand S. A Novel Role for Interleukin-27 (IL-27) as Mediator of Intestinal Epithelial Barrier Protection Mediated *via* Differential Signal Transducer and Activator of Transcription (STAT) Protein Signaling and Induction of Antibacterial and Anti-Inflammatory Protein Signaling and Induction of Antibacterial and Anti-Inflammatory Proteins. J Biol Chem (2012) 287:286–98. doi: 10.1074/jbc.M111.294355 PMC3249079 22069308

[41] Kim G Shinnakasu R Saris CJM Cheroutre H Kronenberg M. A Novel Role for IL-27 in Mediating the Survival of Activated Mouse CD4 T Lymphocytes. J Immunol (2013) . 190:1510–8. doi: 10.4049/jimmunol.1201017 PMC4060896 23335749

[42] Hu S Wong CK Lam CWK. Activation of Eosinophils by IL-12 Family Cytokine IL-27: Implications of the Pleiotropic Roles of IL-27 in Allergic Responses. Immunobiol (2011) . 216:54–65. doi: 10.1016/j.imbio.2010.03.004 20435369

[43] Grainger JR Smith KA Hewitson JP McSorley HJ Harcus Y Filbey KJ. Helminth Secretions Induce *De Novo* T Cell Foxp3 Expression and Regulatory Function Through the Tgf-B Pathway. JEM (2010) 207(11):2331–41. doi: 10.1084/jem.20101074 PMC2964568 20876311

[44] Coakley G McCaskill JL Borger JG Simbari F Robertson E Millar M. Extracellular Vesicles From a Helminth Parasite Suppress Macrophage Activation and Constitute an Effective Vaccine for Protective Immunity. Cell Rep (2017) 19:1545–57. doi: 10.1016/j.celrep.2017.05.001 PMC5457486 28538175

[45] Johnston CJC Smyth DJ Kodali RB White MPJ Harcus Y Filbey KJ. A Structurally Distinct Tgf-B Mimic From an Intestinal Helminth Parasite Potently Induces Regulatory T Cells. Nat Commun (2017) 8:1741. doi: 10.1038/s41467-017-01886-6 29170498 PMC5701006

[46] Bolger AM Lohse M Usadel B. Trimmomatic: A Flexible Trimmer for Illumina Sequence Data. Bioinformatics (2014). 30(15):2114–20 doi: 10.1093/bioinformatics/btu170 PMC4103590 24695404

[47] Morgan M Obenchain V Hayden N. Rsamtools: Binary Alignment (BAM), FASTA, Variant Call (BCF), and Tabix File Import. R Package Version 2.4.0 (2020). Available at: http://bioconductor.org/packages/Rsamtools.

[48] McCarthy DJ Campbell KR Lun ATL Willis QF. Scater: Pre-Processing, Quality Control, Normalisation and Visualisation of Single-Cell RNA-Seq Data in R. Bioinformatics (2017) 33(8):1179–86. doi: 10.1101/069633 PMC5408845 28088763

[49] R Core Team. R: A Language and Environment for Statistical Computing. Vienna, Austria: R Foundation for Statistical Computing (2020). Available at: https://www.R-project.org/.

[50] Lun ATL McCarthy DJ Marioni JC. A Step-By-Step Workflow for Low-Level Analysis of Single-Cell RNA-Seq Data With Bioconductor. F1000Research (2016) 5:2122. doi: 10.12688/f1000research.9501.2 27909575 PMC5112579

[51] Langfelder P Zhang B Horvath S. Dynamictreecut: Methods for Detection of Clusters in Hierarchical Clustering Dendrograms. R Package Version 1 (2016). Available at: https://CRAN.R-project.org/package=dynamicTreeCut.

[52] Zhao S Yin L Guo Y Sheng Q Shyr Y. Heatmap3: An Improved Heatmap Package. R Package Version 1. 1.9 (2021). Available at: https://CRAN.R-project.org/package=heatmap3.

[53] Robinson MD McCarthy DJ Smyth GK. Edger: A Bioconductor Package for Differential Expression Analysis of Digital Gene Expression Data. Bioinformatics (2010) 26:139–40. doi: 10.1093/bioinformatics/btp616 PMC2796818 19910308

[54] Fresno C Fernández EA. Rdavidwebservice: A Versatile R Interface for DAVID. Bioinformatics (2013) 29(21):2810–1. doi: 10.1093/bioinformatics/btt487 23958726

[55] Wu T Wu T Hu E Xu S Chen M Guo P Dai Z. Clusterprofiler 4.0: A Universal Enrichment Tool for Interpreting Omics Data. Innovation (2021) 2(3):100141.55. doi: 10.1016/j.xinn.2021.100141 PMC8454663 34557778

