# Supplementary material for: The Host Peritoneal Cavity Harbors Prominent Memory Th2 and Early Recall Responses to an Intestinal Nematode
Source: Front Immunol. 2022 Mar 28;13:842870. doi: 10.3389/fimmu.2022.842870 (PMC8996181; doi:10.3389/fimmu.2022.842870)
Supplement: Supplementary file 1 [file DataSheet_1.docx]

Supplementary Material


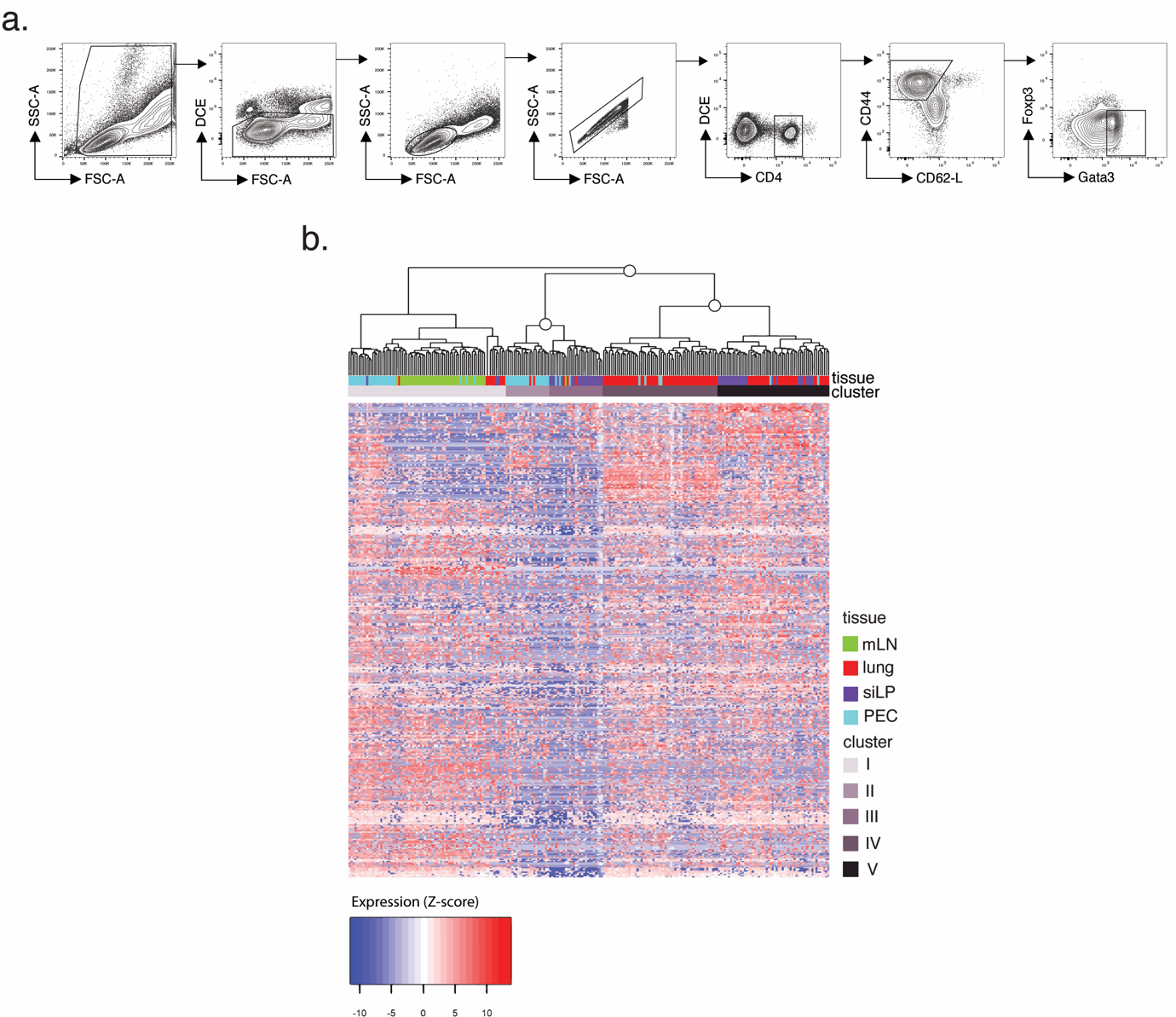


Supplementary Figure 1. (A) Gating strategy for the analysis of memory Th2 cells, gated as live CD4^+^CD44^+^CD62-L^-^Foxp3^-^Gata3^+^. (B) Global heatmap of hypervariable expressed genes over all sorted CD4^+^ T cells from the PEC, siLP, lung and mLN of H. polygyrus-cured mice.


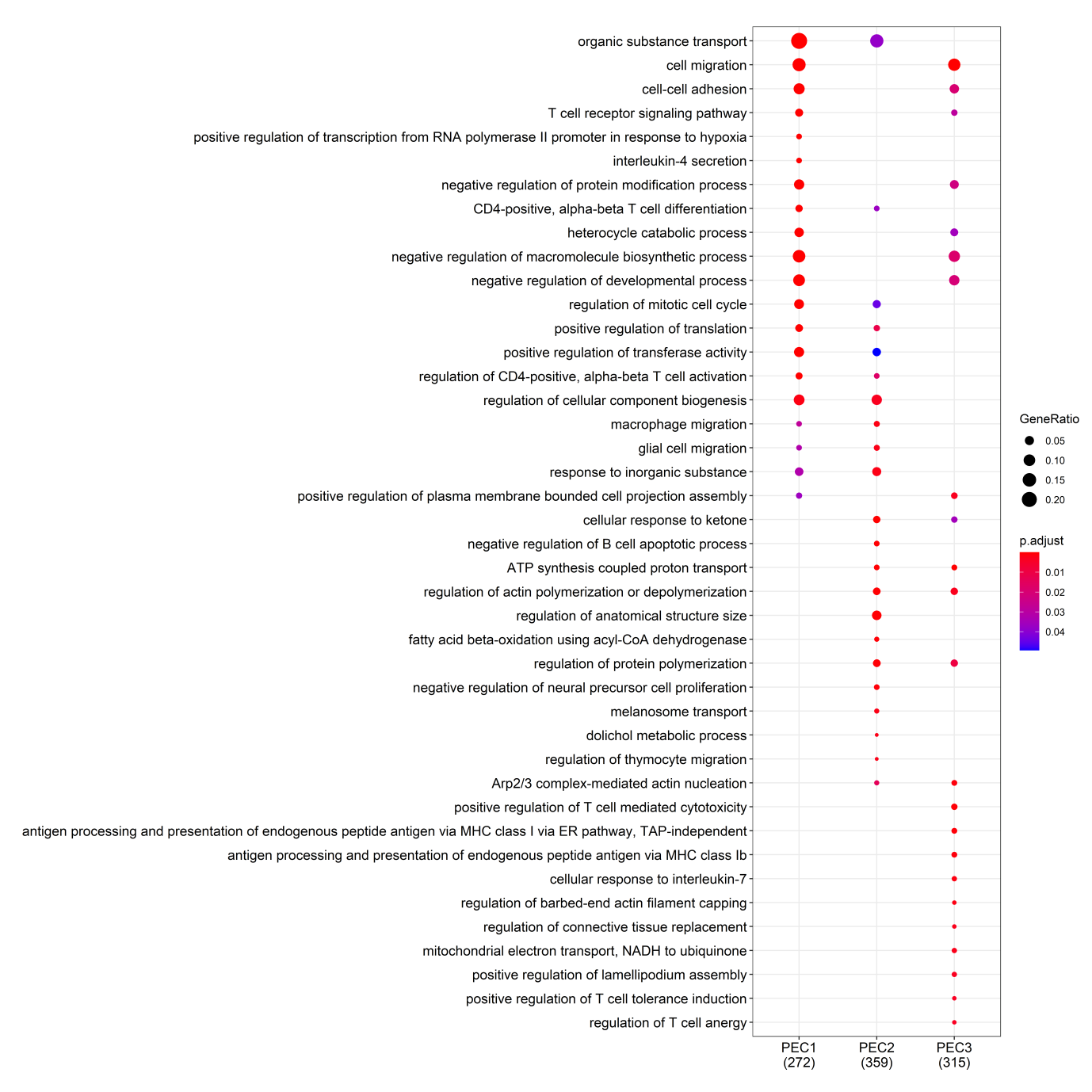


**Supplementary Figure 2.** Results of an overrepresentation analysis of marker genes of the three PEC sub-clusters in the terms of the gene ontology (GO). Terms with significant overrepresentation in all of the three PEC clusters were filtered out. The terms of the remaining results were mapped to the significant most specific term and the top 15 terms for each PEC cluster were compared in the dot plot.


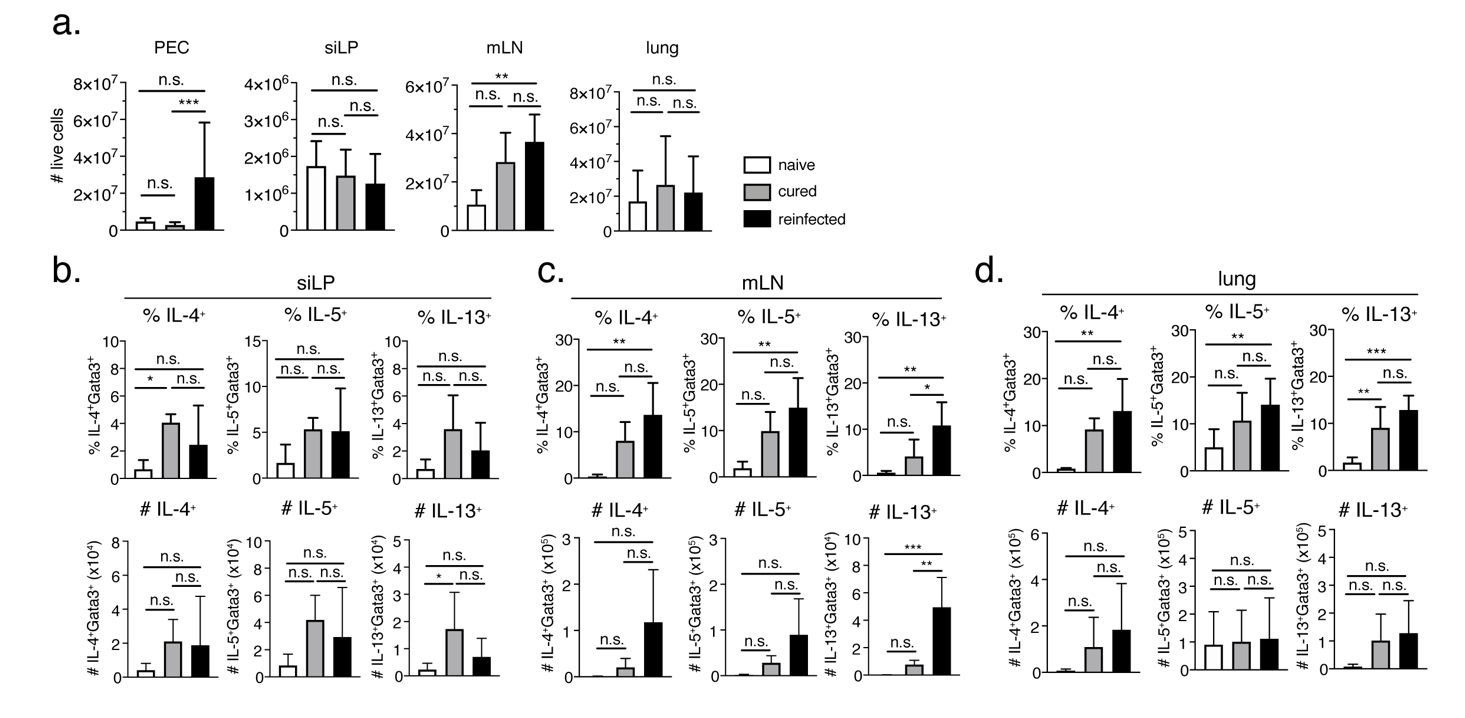


**Supplementary Figure 3.** (A) Absolute numbers of viable cells in PEC, siLP, mLN and lung of naïve, cured and reinfected mice. Frequencies (top panels) and absolute cell numbers (bottom panels) of IL-4^+^, IL-5^+^ and IL-13^+^ Gata3^+^CD4^+^CD44^+^CD62-L^-^Foxp3^-^ memory Th2 cells in the (B) siLP, (C) mLN and (D) lung of naïve, cured and reinfected mice. Statistical analysis was done using a Kruskal- Wallis test combined with Dunn’s multiple comparison test. * p<0.05, ** p<0.01 and *** p<0.001, n.s. not significant.


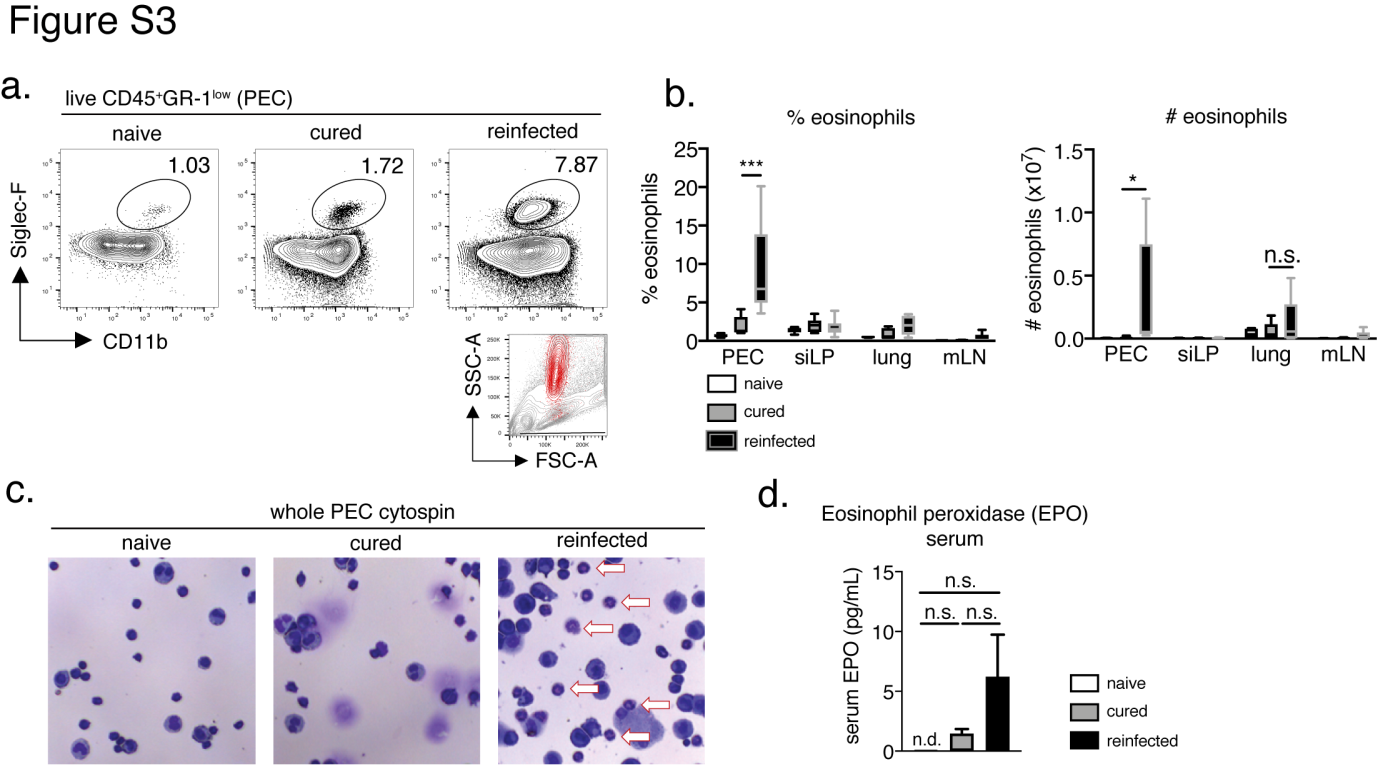


**Supplementary Figure 4.** (A) Exemplary FACS plots showing Siglec-F^+^CD11b^+^GR-1^low^CD45^+^ eosinophil frequencies in the PEC of a naïve, a cured and a reinfected mouse. The red overlay plot below shows the high side scatter (SSC) of the gated eosinophil population, confirming their typically high granularity. (B) Frequencies and absolute numbers of eosinophils in PEC, siLP, mLN and lung of naïve, cured and reinfected mice. (C) Exemplary cytospins of whole PEC samples from a naïve, a cured and a reinfected mouse. The white arrows indicate peritoneal eosinophils. (D) Eosinophil peroxidase levels in serum from naïve, cured and reinfected mice. The data are pooled from two independent experiments with n=3-5 mice per group. Statistical analysis was done using one-way ANOVA combined with Tukey’s multiple comparison test. * p<0.05, *** p<0.001, n. s. not significant, n.d. not detected.


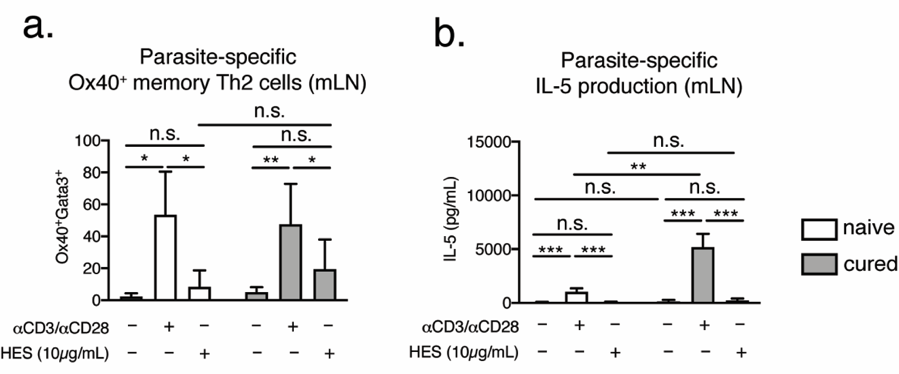


**Supplementary Figure 5.** (A) Frequencies of Ox40^+^ in Gata3^+^CD44^+^CD62-L^-^Foxp3^-^ memory Th2 cells following a 72-hour *in vitro* restimulation with αCD3/CD28 monoclonal antibodies and *H. polygyrus* excretory/secretory antigen (HES, 10 µg/mL) of whole mLN cells from naïve and cured mice. (B) IL-5 cytokine levels in cell culture supernatants following the *in vitro* restimulation of whole mLN cells described in (A). The data are pooled from two independent experiments with n=2-3 mice per group. Statistical analysis was done using a Kruskal-Wallis test combined with Dunn’s multiple comparison test. * p<0.05, ** p<0.01 and *** p<0.001, n.s. not significant.


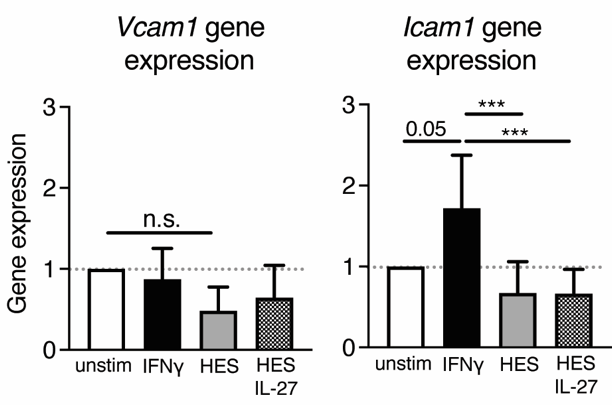


**Supplementary Figure 6.** Gene expression levels of *Vcam1* and *Icam1* in peritoneal membrane snips following *in vitro* restimulation with IFNγ (10ng/mL), HES (10µg/mL) or HES and IL-27 (10µg/mL and 50ng/mL, respectively) against GAPDH as a housekeeping gene and normalized against unstimulated controls. The data are pooled from two independent experiments with n=2-4 mice per group. Statistical analysis was done using one-way ANOVA combined with Tukey’s multiple comparison test. * p<0.05, ** p<0.01, *** p<0.001, n.s. not significant.

**Supplementary Table 4.** Antibodies used for flow cytometry analysis of surface and intracellular markers.

| **Marker** | **Antibody clone** | **Fluorochrome** | **Commercial supplier** |
| --- | --- | --- | --- |
| CD4 | RM4-5 | A700 | BD |
| CD4 | RM4-5 | PerCP | BioLegend |
| CD44 | IM7 | eF450 | Thermo Fisher Scientific |
| CD45 | 30-F11 | PE-Cy7 | Thermo Fisher Scientific |
| CD45.2 | 104 | A700 | Thermo Fisher Scientific |
| CD62-L | MEL-14 | APC-Cy7 | Thermo Fisher Scientific |
| CD69 | H1.2F3 | PE-Cy7 | Thermo Fisher Scientific |
| Ox40 | OX-86 | BV605 | BioLegend |
| Foxp3 | FJK-16s | eF610 | Thermo Fisher Scientific |
| Gata3 | TWAJ | eF660 | Thermo Fisher Scientific |
| IL-4 | 11B11 | BV421 | BioLegend |
| IL5 | TRFK5 | PE | Thermo Fisher Scientific |
| IL-13 | eBio13A | A488 | Thermo Fisher Scientific |
| Siglec-F | E50-2440 | PE | BD |
| CD11b | M1/70 | FITC | BD |
| Ly6G | 1A8-Ly6G | eF450 | Thermo Fisher Scientific |
| CD11c | N418 | eF450 | Thermo Fisher Scientific |
| MHC-II | M5/114.5.2 | Pe-Cy5 | Thermo Fisher Scientific |
| Ox40L | RM134L | APC | Thermo Fisher Scientific |

| **Target** | **Forward primer** | **Reverse primer** |
| --- | --- | --- |
| ICAM-1 | 5′-CAATTTCTCATGCCGCACAG-3′ | 5′-AGCTGGAAGATCGAAAGTCCG-3′ |
| VCAM-1 | 5′-TGAACCCAAACAGAGGCAGAGT-3′ | 5′- GGTATCCCATCACTTGAGCAGG-3′ |
| GAPDH | 5’-GCACTTGGCAAAATGGAGAT-3’ | 5’-CCAGCATCACCCCATTAGAT-3’ |

**Supplementary Table 5.** Primers used for quantitative real-time PCR (qRT-PCR) analysis.
